# Computational pathology-based weakly supervised prediction model for MGMT promoter methylation status in glioblastoma

**DOI:** 10.3389/fneur.2024.1345687

**Published:** 2024-02-07

**Authors:** Yongqi He, Ling Duan, Gehong Dong, Feng Chen, Wenbin Li

**Affiliations:** ^1^Department of Neuro-Oncology, Cancer Center, Beijing Tiantan Hospital, Capital Medical University, Beijing, China; ^2^Department of Pathology, Beijing Tiantan Hospital, Capital Medical University, Beijing, China

**Keywords:** computational pathology, glioblastoma, deep learning, MGMT, diagnostic

## Abstract

**Introduction:**

The methylation status of oxygen 6-methylguanine-DNA methyltransferase (MGMT) is closely related to the treatment and prognosis of glioblastoma. However, there are currently some challenges in detecting the methylation status of MGMT promoters. The hematoxylin and eosin (H&E)-stained histopathological slides have always been the gold standard for tumor diagnosis.

**Methods:**

In this study, based on the TCGA database and H&E-stained Whole slide images (WSI) of Beijing Tiantan Hospital, we constructed a weakly supervised prediction model of MGMT promoter methylation status in glioblastoma by using two Transformer structure models.

**Results:**

The accuracy scores of this model in the TCGA dataset and our independent dataset were 0.79 (AUC = 0.86) and 0.76 (AUC = 0.83), respectively.

**Conclusion:**

The model demonstrates effective prediction of MGMT promoter methylation status in glioblastoma and exhibits some degree of generalization capability. At the same time, our study also shows that adding Patches automatic screening module to the computational pathology research framework of glioma can significantly improve the model effect.

## 1 Introduction

Glioma is the most prevalent primary malignant tumor in the central nervous system. Glioblastoma (GBM) is the most common glioma subtype and an important cause of morbidity and mortality. It progresses rapidly and has the worst prognosis with a 5-year survival rate of less than 7% (1). For individuals newly diagnosed with GBM, the current standard treatment remains gross total resection followed by a combination of radiation therapy and temozolomide (TMZ) (2). O6-methylguanine–DNA methyltransferase (MGMT) is a DNA repair enzyme that reverses the DNA damage caused by alkylating agents, resulting in tumor resistance to TMZ and nitrosourea-based systemic therapy. Epigenetic silencing of the MGMT gene by promoter methylation makes the tumor more sensitive to treatment with alkylating agents and has been associated with longer overall survival in patients with GBM who received TMZ chemotherapy (3). There are multiple ways to detect MGMT promoter methylation, including methylation-specific PCR, methylation-specific high-resolution melting, and pyrosequencing. However, these methods are often time-consuming and labor-intensive, and not all patients have the conditions to undergo relevant examinations. Obtaining an accurate diagnosis swiftly remains a significant challenge (4).

Histopathological biopsy, particularly hematoxylin and eosin (H&E)-stained slides, is still a gold standard for tumor diagnosis (5). However, accurate diagnosis of tumors requires high professionalism from pathologists. The clinical experience and subjectivity of the pathologist can also affect the diagnostic results (6). With the advancement of digital imaging and computer technology in recent years, computational pathology based on whole slide images (WSI) for artificial intelligence (AI)-assisted analysis is rapidly developing (7). For example, computational pathology can predict tumor classification (8, 9), prognosis (10), and molecular mutations based on histopathological images, etc. (11, 12). However, as far as we know, research regarding MGMT promoter methylation in glioma using pathological images remains relatively limited. Li introduced a weakly supervised primary brain tumor classifier VIT-WSI based on digital pathology slides. Within this framework, the MGMT promoter methylation status of gliomas was predicted with an accuracy of 0.7916 and an AUC of 0.845 (13). However, it is essential to note that the primary focus of the study was not on the prediction of MGMT promoter methylation. This study included only 71 patients with known MGMT methylation status, and 53 of those were determined by immunohistochemistry (IHC). Though IHC is a common and inexpensive assay used in clinical, the value of using IHC to determine MGMT status has been controversial. Due to the inconsistency between MGMT promoter methylation status and MGMT protein expression, the IHC results may not be accurate, thus affecting patients’ treatment and prognosis (4). This indicates that it is of great clinical significance for us to develop a novel predictive model for the MGMT promoter methylation status. A study published in 2022 by Kim et al. (14) showed that MGMT promoter methylation was significantly associated with ATRX gene deletion in IDH wild-type glioblastoma. Moreover, ATRX is involved with the telomerase-independent alternative lengthening of telomeres (ALT) mechanism, and the ATRX gene is also involved in the regulation of the tumor microenvironment in glioma (15). Therefore, we hypothesized that MGMT promoter methylation in glioblastoma may cause subtle changes in tumor cell morphology through interactions with other genes, which can be captured by the neural network model to obtain corresponding predictions. Hence, we proposed a weakly supervised prediction model for MGMT promoter methylation status in glioblastoma based on computational pathology with H&E-stained histopathological slides. We used two models based on transformer architecture to achieve the end-to-end prediction of MGMT promoter methylation status and confirmed the generalization of the overall model using datasets from different sources. The contribution of this study is 2-fold: one is to confirm the possibility of predicting the methylation status of MGMT promoter in gliomas by WSI; second, an end-to-end model was established to simplify the cost of MGMT promoter status detection in glioma patients.

## 2 Materials and methods

### 2.1 Dataset

At present, the existence of an open database is very important. The complexity of cancer disease requires a large amount of data as a supporting basis, and the existence of an open database can accelerate related research and save researchers’ resources (16). Therefore, this retrospective study included histopathological slides of H&E-stained tissue samples from two cohorts of glioblastoma patients. The first cohort comprised patients from The Cancer Genome Atlas (TCGA) project, accessible through the National Institutes of Health Genome Data Sharing Portal (17–19) (TCGA cohort). The second cohort comprised patients with glioblastoma diagnosed in Beijing Tiantan Hospital from 2019 to 2023 (TianTan cohort). As cryopreservation destroys the original morphological structure of cells and tissues, all histopathological slides collected in this study were preserved by formalin-fixed paraffin embedding. Specific data inclusion and exclusion criteria are listed below:

Inclusion criteria were as follows: (1) precise diagnosis of glioblastoma by surgical pathology; (2) clear methylation status of the MGMT promoter by genetic testing; and (3) no other brain lesions. Exclusion criteria were as follows: (1) unclear images of histopathological slides; and (2) missing baseline information or unknown medical history.

All pathological images in the TCGA cohort were confirmed to be glioblastoma by secondary diagnosis by a pathologist with extensive experience in pathological diagnosis. All histopathological slides in the TianTan cohort were scanned using a Lecia Aperio CS2 scanner into WSIs of digital pathology in standard file format (the “svs” format). The scanning magnification was 20X. All data from the TianTan cohort were only used as an external independent test set to verify the generalization of the model and were not used in the training or hyperparameter adjustment process. Data in the TCGA cohort were divided into the training set and the test set according to certain standards: If multiple WSIs originated from the same patient, then all those WSIs from that patient were assigned to the training set. This approach ensured that slides from the same tumor sample were not used for both training and testing at the same time.

This study was approved by the Ethics Committee of Beijing Tiantan Hospital (ethical approval no: YW2022-025).

### 2.2 Overall model architecture

**Figure 1:**
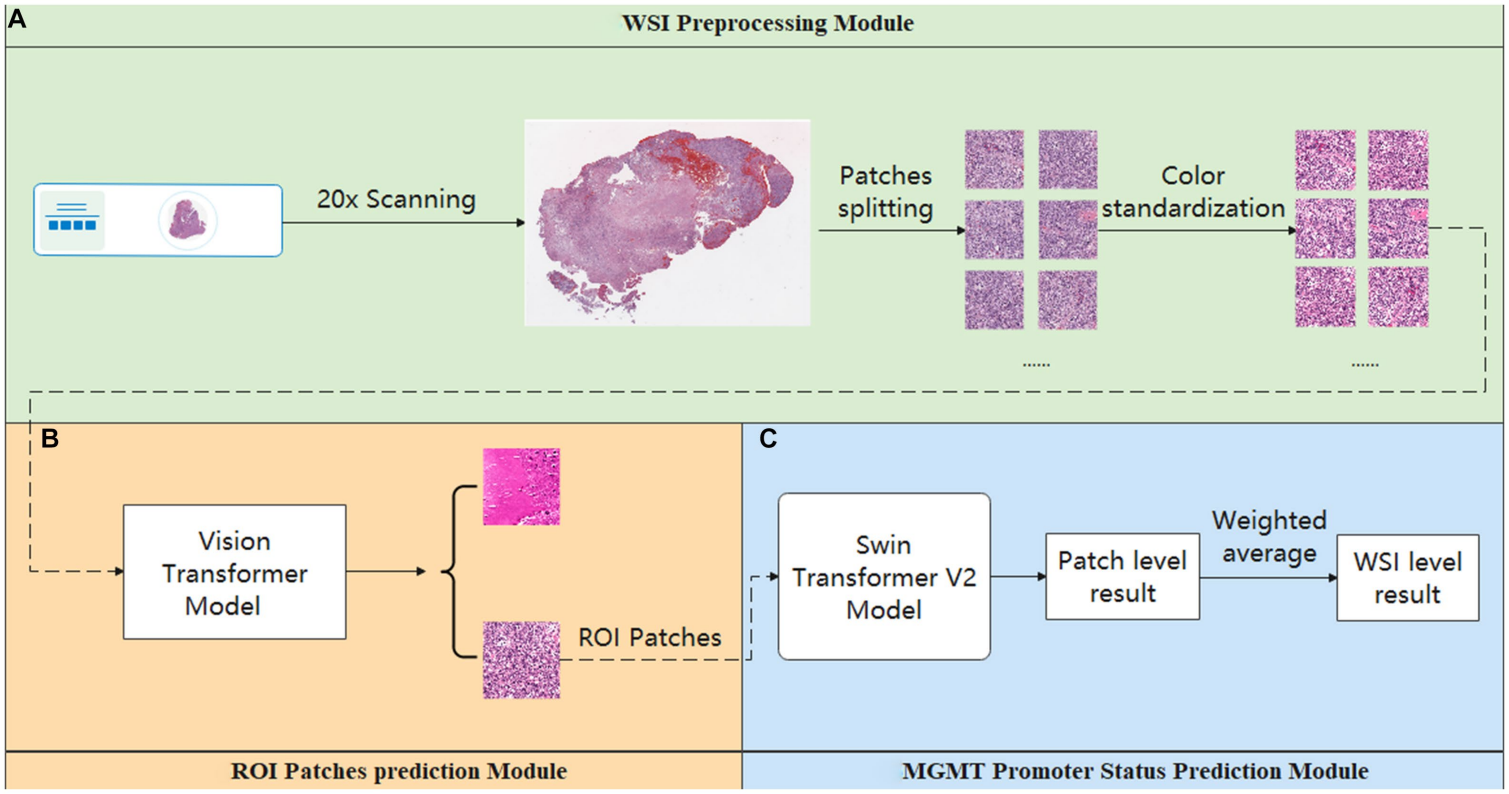
Overview process of the model. **(A)** Preprocessing module: responsible for data pre-processing. **(B)** ROI patches prediction module: responsible for automatically annotating ROI patches. **(C)** MGMT promoter status prediction module: responsible for acquiring the patient’s MGMT prediction results.

Our study achieved end-to-end prediction of MGMT promoter methylation status in glioma patients based on H&E-stained WSIs through three main modules. First, the scanned WSIs were divided into several patches using the preprocessing module. After that, the glioblastoma region of interest (ROI) patches prediction module was used to filter the sliced patches. Finally, the molecular status of patients was predicted by the MGMT promoter status prediction module. The main body model framework adopts the modular design of openMMLab (20). The details are described below. The complete process diagram is shown in Figure 1.

### 2.3 Preprocessing module

This module is crucial for pre-processing WSIs. It consisted of two parts. The first part is the patch segmentation. This part is responsible for splitting the complete WSI into patches. A complete WSI consists of billions of pixels through a pyramid structure. It is difficult to directly input such large image data into a neural network or machine learning model for training. Therefore, we used the sliding window method to divide the tissue image in WSI into several patches of 512 pixels × 512 pixels for subsequent data analysis and processing (21). The second part is the color normalization. This part is dedicated to the color normalization of the patches. Color variation can arise due to several factors, including variations introduced by the operator during the H&E staining and differences in staining reagents. Such color variations can significantly interfere with subsequent modeling predictions. Thus, we used the Vahadane method to normalize the colors presented in H&E pathology images to eliminate the influence of staining differences, ensuring consistent and reliable data for further analysis (22).

### 2.4 ROI patches prediction module

This module is used to screen the patches obtained in the Preprocessing module. As a WSI may encompass diverse elements such as hemorrhage and normal tissue, potentially interfering with the final prediction, we employed the ROI patches prediction model to screen the patches generated in the preprocessing module to avoid the interference of confusing information as much as possible. The module utilized transfer learning, employing a Vision Transformer model pre-trained on the ImageNet dataset (23). We optimized the model’s performance using the AdamW optimizer (24). To further enhance model convergence during training, we employed the CosineAnnealingLrUpdater strategy for optimal learning rate adjustments (25). The initial learning rate is set at 1.75 × 10^−3^, with a minimum of 1.75 × 10^−5^ imposed during the learning rate adjustment process. We also employed linear preheating for the learning rate. Model training and prediction used the LabelSmoothLoss as the loss function (26).

In this process, the training data for the Vision Transformer model were derived from the annotations of pathologists. Additionally, pathologists screened patches to ensure that the training dataset exclusively includes ROI patches exhibiting typical tumor characteristics.

In addition, to investigate the potential impact of refined annotation on the results, we explored other two methods to filter the patches generated by the Preprocessing Module. This included relying on the pathologist’s annotation for filtering (Tumor Only) and only removing the blank background (Whole Tissue). The implementation details for each method are described below:

1. Tumor only: For this method, two highly experienced pathologists annotated ROI regions. When the annotated ROI regions were inconsistent, a third more senior pathologist made the final judgment. When screening the patches, if a patch does not completely exist in the ROI area, we will discard it. Only patches fully contained within the ROI area will be retained.
2. Whole tissue: In this approach, while screening the patch, we first evaluate its overall color threshold. Given the RGB color distribution ranging from 0 to 255, where values closer to 255 represent colors closer to white. Therefore, patches with an overall RGB value greater than 220 were considered blank backgrounds and were discarded. The rest of the patches were retained for further analysis.

Through the above method, we obtained three groups of patches annotated in different ways. These three groups of patches were input into the final MGMT Predict Module to obtain diverse results and to compare the impact of these three annotation methods on the results.

### 2.5 MGMT promoter status prediction module

The ROI patches output by the second module were used as the input of this module to predict the final MGMT promoter methylation status. The processing flow of this module involved considering each WSI as a package, which is then sliced into numerous ROI patches. Each ROI patch is treated as an instance, with the package’s label being assigned to each patch within it. In this part, the main structure of our model adopts the Swin Transformer v2 structure (27). After position encoding of input data using PatchEmbed, the four stages are passed. Except for the first stage, each stage consists of a PatchMerging module and several Swinblock V2 modules. Then, the features are processed by the linear normalization layer and global average pooling layer and then predicted and classified by a fully connected layer. Each SwinBlockV2 module consists mainly of multi-head attention mechanisms with feedforward neural networks and linear normalization layers. In the four stages, the number of SwinBlockV2 is [2, 2, 18, 2], respectively, and the number of attention heads for each SwinBlockV2 is [3, 6, 12, 24], respectively. In this way, for each WSI, we could get the prediction probability of several patches and perform a weighted average of all the prediction results to obtain the final patient-level prediction. This ensured that each patch’s predictions contributed appropriately to the patient-level result. The weighting formula was as follows:


$${\mathrm{Weight}}_{i}=\frac{\mathrm{|pred}\_{\mathrm{score}}_{i}-0.5|}{\sum_{i=1}^{n}|\mathrm{pred}\_{\mathrm{score}}_{i}-0.5|}$$

$\mathrm{Weigh}t_{i}$ represents the weight that the prediction result of the *i*-th patch contributes to the final weighted average, and $\mathrm{pred}\_\mathrm{scor}e_{i}$ signifies the prediction result of the i patch.

For patch-level results, weights were assigned based on the degree of deviation of their prediction probability from 0.5. This strategic weighting reduced the influence of results with prediction probabilities near 0.5 on the final average, thereby strengthening the reliability of model predictions.

### 2.6 Statistical analysis

We used SPSS 26 to analyze the differences in clinical baseline data of patients using a *t*-test and chi-square test. Python (3.7.9) was used for data processing and analysis and overall model construction. We employed the PyTorch framework to construct the neural network, and the hardware platform for model training was the NVIDIA A40 GPU with 48G of memory.

## 3 Results

### 3.1 Clinical baseline characteristics

According to the inclusion and exclusion criteria, we included 29 WSIs from 29 patients in the TianTan cohort and 632 WSIs from 199 patients in the TCGA cohort. A total of 57 patients in the TCGA database had only one WSI based on the previously described criteria, so 57 WSIs from these 57 patients were used as an independent test set for the TCGA cohort. The remaining 575 WSIs from 142 patients were used as the training set. According to the fifth edition of the WHO Classification of Tumors of the Central Nervous System (WHO CNS5) (5), we excluded patients with IDH mutation from the test set. Therefore, 25 patients with corresponding 25 WSIs were finally included in the TianTan cohort.

There were no statistically significant differences between the two cohorts regarding age, sex, and MGMT promoter methylation status. Basic clinical characteristics are presented in Table 1.

**Table 1:** Clinical and demographic characteristics of patients.

	TianTan (*n* = 25)	TCGA (*n* = 199)	*p* value
Age, mean ± SD (years)	58.40 ± 12.50	57.24 ± 14.39	0.365
Sex			0.822
Male	15 (60%)	121 (61%)	
Female	10 (40%)	78 (39%)	
MGMT promoter status			0.167
Methylated	13 (52%)	75 (38%)	
Unmethylated	12 (48%)	124 (62%)	

### 3.2 Model training and evaluation results

Our primary evaluation metrics included accuracy and the area under the curve (AUC) value of the receiver operating characteristic (ROC) curve. These metrics were used to assess the performance of the models in both the glioblastoma ROI patches prediction module and the MGMT promoter status prediction module.

**Figure 2:**
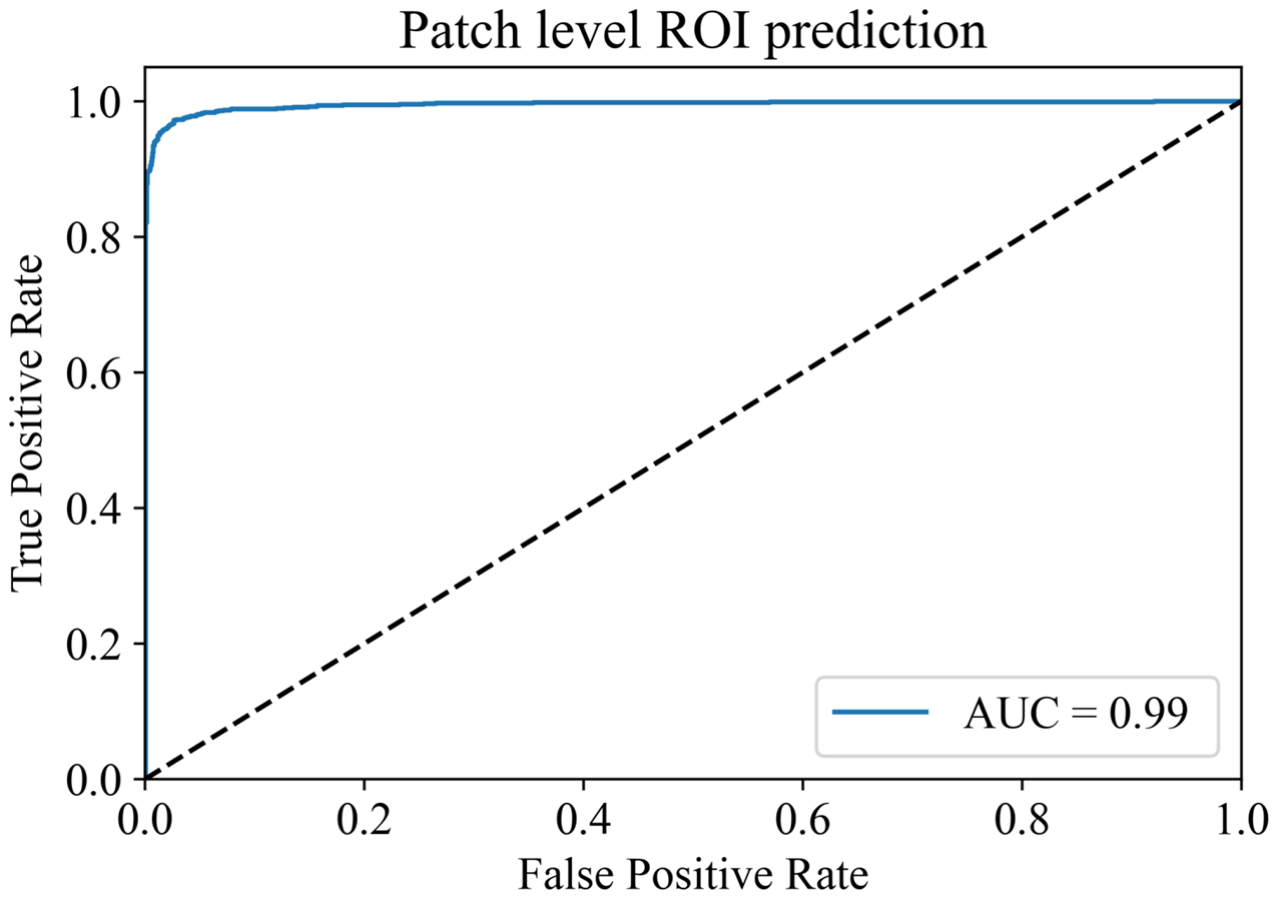
Patch-level ROI prediction ROC curve.

In the ROI patches recognition module, the training dataset comes from the 100 WSIs in the training set of the TCGA cohort described earlier. After segmentation and color normalization, 14,000 patches with typical characteristics were selected under the guidance of professional pathologists. There were 7,000 patches each in the ROI area and non-ROI area. The dataset was divided into training, validation, and test sets in a 6:2:2 ratio. During training, a batch size of 224 and 100 epochs were used for the parameter settings of the model. The model reached the highest accuracy of 97.88% in the validation set at the 80th epoch, and the model parameters at this time were saved for testing in the test set. The final accuracy of the ROI patches prediction module in the test set is 97.03%, and the AUC value is 0.9940 (Figure 2). The results show that the module has a superior ability to identify ROI patches, which can meet the screening needs of the subsequent modules.

In the MGMT promoter status prediction module, we first used the Swin Transformer V2 model for training. The ROI patches output by the ROI patches prediction module was used as the training data of this module. Whether the patient’s MGMT promoter was methylated or not was taken as the label of the patches cut out from that patient. Therefore, we finally obtained 59,945 MGMT promoter methylated patches and 97,431 MGMT promoter unmethylated patches.

**Figure 3:**
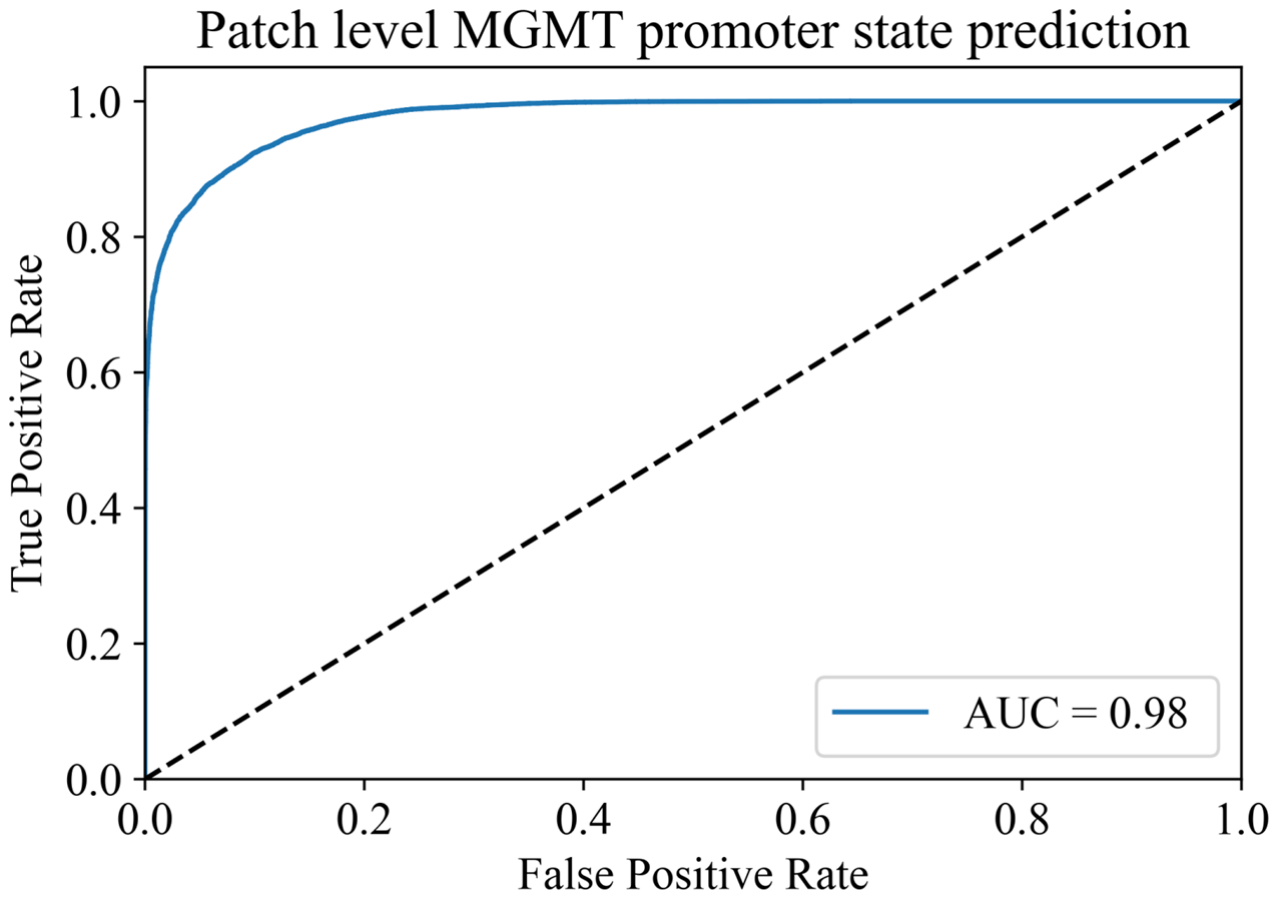
Patch-level MGMT promoter status prediction ROC curve.

To address the issue of category imbalance, we employed random rotation and mirroring to expand both categories to 100,000, with a total of 200,000 patches for model training. The dataset was divided into training, validation, and test sets in a 6:2:2 ratio. We set the batch size to 148 for training parameters and the number of epochs to 500. Remarkably, during the 493rd epoch, the model achieved its highest accuracy of 91.38% in the validation set. We saved the model weights at this time for testing on the test set. In the end, the model exhibited an accuracy of 91.12% in the test set, with an AUC of 0.98 (Figure 3). At the same time, we compared it with the current commonly used Resnet50 model, and the comparison results are shown in Table 2. These results show the ability of the model to accurately distinguish whether the MGMT promoter is methylated at the patch level.

**Table 2:** Comparison between the Swin transformer V2 and Resnet50.

Model	Accuracy	Precision	Recall	F1 score	AUC
Swin_trans_V2	0.91	0.92	0.91	0.91	0.98
Resnet50	0.72	0.74	0.70	0.72	0.80

**Figure 4:**
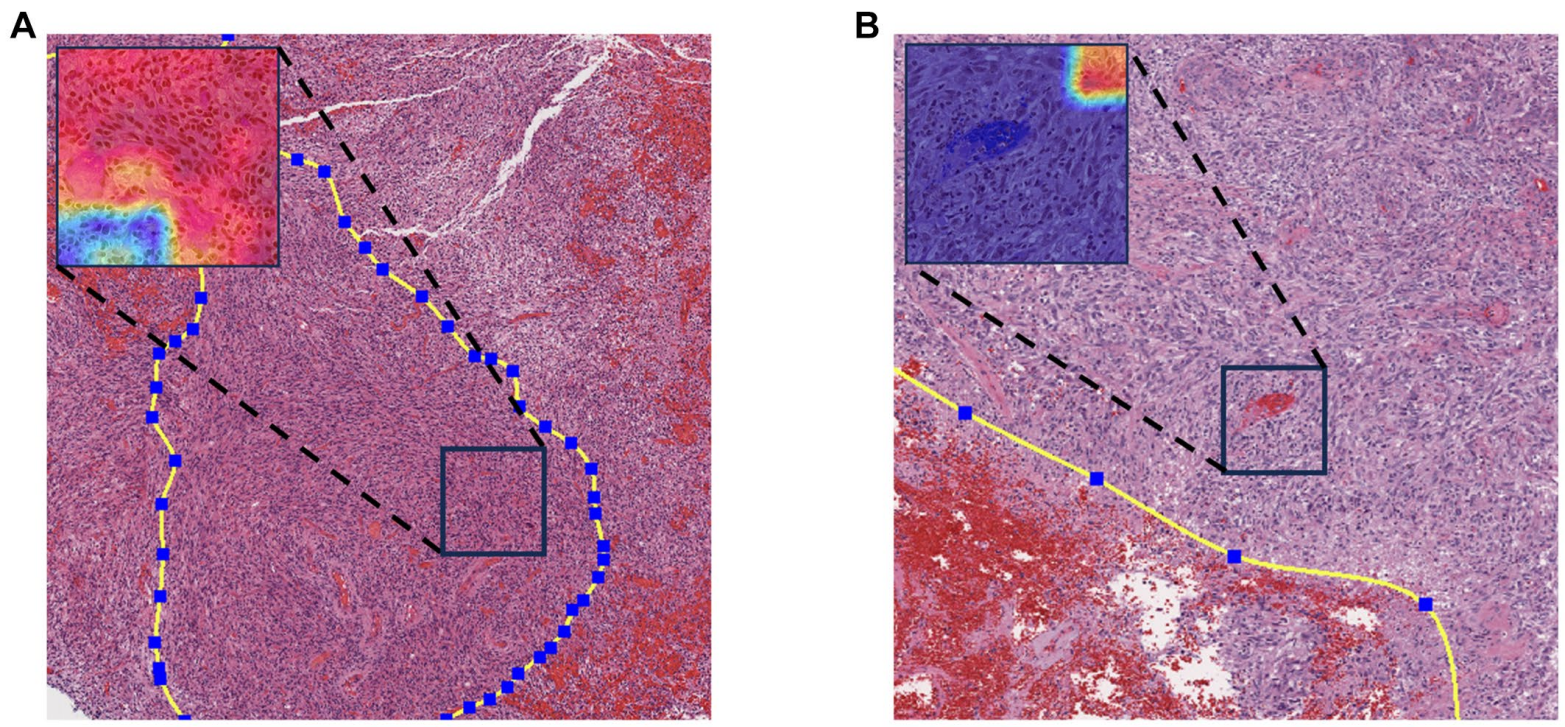
Gradient-weighted class activation mapping. Redder colors in the graph indicate a greater likelihood of methylation. **(A)** WSI with MGMT promoter methylation. **(B)** MGMT promoter unmethylated WSI.

To enhance the model’s interpretability, we utilized a class gradient activation heatmap to visualize the model’s recognition of patches. In this visualization, regions with a redder color indicate a higher probability that those areas are methylated. As depicted in Figure 4A, represents a WSI with MGMT promoter methylation, while Figure 4B represents a WSI without MGMT promoter methylation. Each image’s upper left corner displays the corresponding patch’s gradient class activation heatmap. The results demonstrate that the models correctly identify their respective patches.

Hence, we aimed to extrapolate the patch-level results to the patient level. The patches obtained by three different methods were input into the MGMT promoter status prediction module for testing. According to the ROC curve of previous patch-level evaluation, we calculated that the best cutoff value for judging whether MGMT promoter methylation exists at the patch level was 0.52; that is, when the probability of methylation of a patch MGMT promoter judged by the model was greater than 0.52, we think it has MGMT promoter methylation. We use this cutoff value for patient-level model evaluation. To obtain patient-level results, we aggregated the patch-level outcomes using the probability-weighted average method as previously described. The results are presented in Table 3.

**Table 3:** Patient-level test results.

	Tumor only	Whole tissue	Module predict
TCGA Test (*n* = 57)			
Balance_Accuracy	0.79	0.75	0.79
AUC	0.81	0.84	0.86
Recall	0.84	0.74	0.74
Specificity	0.74	0.76	0.84
Precision	0.62	0.61	0.70
F1 score	0.71	0.67	0.72
TianTan Test (*n* = 25)			
Balance_Accuracy	0.76	0.69	0.76
AUC	0.75	0.69	0.83
Recall	0.69	0.46	0.69
Specificity	0.83	0.92	0.83
Precision	0.82	0.86	0.82
F1 score	0.75	0.60	0.75

**Figure 5:**
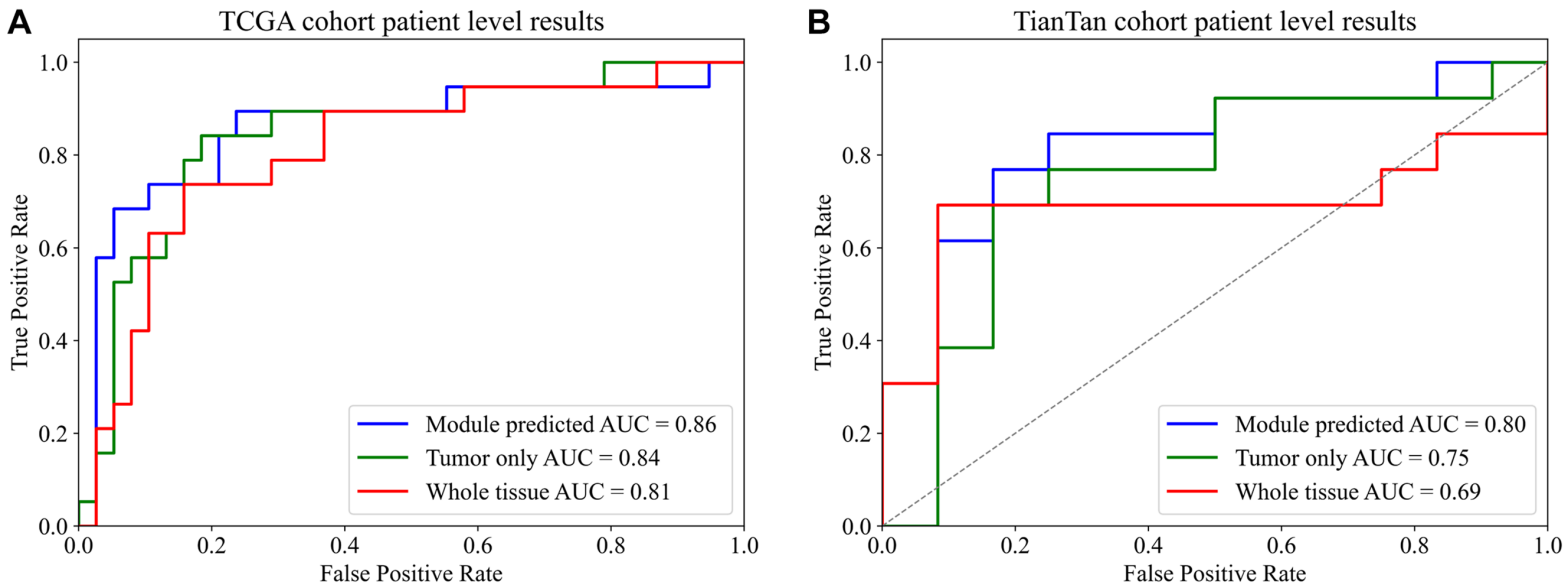
Final patient-level ROC curve. **(A)** TCGA cohort patient-level results. **(B)** TianTan cohort patient-level results.

In both TCGA and TianTan test sets, the approach combining the ROI patches prediction module with the MGMT promoter status prediction module demonstrates the highest AUC values. Moreover, the approach combining the ROI patches prediction Module with the MGMT promoter status in the Tiantan cohort and the TCGA cohort T achieved the same highest balance accuracy. The ROC curves for these three methods in the test set are depicted in Figure 5. These comprehensive results demonstrate that the overall process we designed can effectively determine the presence or absence of MGMT promoter methylation in patients through the H&E-stained histopathological slides.

## 4 Discussion

In this study, we introduced a weakly supervised deep learning model based on H&E-stained histopathological slides to determine the MGMT promoter methylation status of glioblastoma. The methylation status of MGMT gene promoter has a significant impact on histological typing and diagnosis as well as predicting patient survival and response to treatment. Although there is much evidence that it plays an important role in prognosis and treatment, its routine implementation in clinical practice has been challenging (3, 28). The best method and optimal cutoff definitions for MGMT status determination remain under debate. Thus, there is an urgent need to explore a simpler and faster way to obtain the status of the MGMT promoter.

Current research on AI-assisted prediction of MGMT promoter methylation in glioma mainly focuses on radiomics. However, the efficacy of MRI imaging measurement indicators to predict the methylation status of the MGMT promoter remains controversial. For instance, Choi et al. (29) reported that there was no significant difference between the apparent diffusion coefficient (ADC) values and MGMT promoter methylation status. This finding is consistent with the latest research results of Ma et al. (30), which suggest that specific MRI measures such as ADC may not reliably distinguish from MGMT promoter methylation status. In contrast, a study conducted by Han et al. in 2018 reported different findings. They observed a significant increase in ADC values in preoperative MRI scans of primary glioblastoma with MGMT promoter methylation. Furthermore, they utilized this difference as a classification predictor, achieving an AUC value of 0.86 (31). Indeed, machine learning and deep learning approaches have gained significant attention in recent years for predicting MGMT promoter methylation status in glioblastoma. Pasquini et al. (32) employed a machine learning approach to forecast the methylation status of the MGMT promoter in glioblastoma patients using MRI, achieving an accuracy of 70.8% and an AUC value of 0.688. Chen et al. (33) extracted MRI-related features from diffuse gliomas and utilized a ResNet network for training. The model achieved an impressive AUC value of 0.90 and an accuracy of 91.0% in the test set (33). Therefore, we believe that more innovative and effective methods and approaches for MGMT promoter methylation prediction models are imperative. Our investigation employed two neural networks with transformer-like architectures to predict the MGMT promoter methylation status in glioblastoma. Our findings align with Li′s study and prove the feasibility of utilizing histopathological images for MGMT promoter methylation status prediction (13). At the same time, we noticed that some recent studies used HE-stained pathological sections to predict MGMT promoter methylation. For example, Mili et al. (34) proposed a MobileNetV2 model that introduced a spatial attention correlation mechanism to predict grade II and III gliomas, but their results only stayed at the patch level. No predictions were made for the patients. In addition, the study by Krebs et al. (35) also proposed the method of comparing self-supervised learning (SSL) and dual-stream multi-instance learning (DSMIL) to predict the methylation state of MGMT promoter by using HE-stained slices and achieved good results. Our research further suggests that adding an automatic data screening module in the process of model training and prediction may achieve better results.

In future research, WSI, as a different kind of data from MRI, may exhibit more accurate predictions of the molecular status of brain tumors. The large amount of information carried by WSI is more suitable for the use of deep learning models. Furthermore, the inherently two-dimensional characteristics of WSI offer more opportunities for widely interdisciplinary collaboration with the latest image classification algorithms in the computer field, such as transformer-based architectures. Additionally, the proposal of advanced deep learning techniques such as multiple instances learning and graph neural networks has further promoted the development of deep learning based on WSI (36, 37). Especially when WSI and MRI are combined, it may produce remarkable prediction effects in building brain tumor classification prediction models. However, it is also worth noting that the results obtained from simple MRI data and pathological section images are not enough to be directly applied to clinical applications (38). In the future, we must incorporate more clinical and even genetic characteristics of patients to build a multi-dimensional predictive model. To improve the reliability of our results. In addition, the disadvantage of the current deep learning model is that it is easy to become a black box. We do not know what features in the pathological sections make the model final, which is a problem for clinicians. To solve the problem of explainability of models, the development of explainable computational pathology combined with explainable AI in recent years may be an important direction for future research (39).

Indeed, this study has some limitations, including the relatively limited samples from Beijing TianTan Hospital and the utilization of data from a single source. In the future, we will try to incorporate more sources of data for analysis and combine MRI data to build multimodal models to further enhance the accuracy and robustness of predictions.

## 5 Conclusion

Our weakly supervised prediction model based on H&E-stained histopathological slides proves to be an effective approach for predicting the MGMT promoter methylation status in glioblastoma. Additionally, accurate annotation of the tumor region can significantly enhance the predictive performance of the model.

## Data availability statement

The original contributions presented in the study are included in the article/supplementary material, further inquiries can be directed to the corresponding author.

## Ethics statement

The studies involving humans were approved by the Institutional Review Board of Beijing Tiantan Hospital, Capital Medical University. The studies were conducted in accordance with the local legislation and institutional requirements. Written informed consent for participation was not required from the participants or the participants’ legal guardians/next of kin because this is a retrospective study and only the patient’s previous pathological image information was used.

## Author contributions

YH: Conceptualization, Formal analysis, Methodology, Project administration, Writing – original draft. LD: Data curation, Investigation, Writing – review & editing. GD: Resources, Writing – review & editing. FC: Investigation, Writing – review & editing. WL: Conceptualization, Supervision, Writing – review & editing.
